# Effects of *Yucca schidigera* Supplementation on In Vitro Cecal Fermentation and In Vivo Nutrient Digestibility in Male and Female Lean Fattening Pigs

**DOI:** 10.3390/ani16091354

**Published:** 2026-04-28

**Authors:** Matteo Santoru, Jennifer Muñoz-Grein, María Ángeles Latorre, Luciano Pinotti, Luciana Rossi, Javier Alvarez-Rodriguez

**Affiliations:** 1 Department of Veterinary Medicine and Animal Sciences (DIVAS), Università degli Studi di Milano, 26900 Lodi, Italy; luciano.pinotti@unimi.it (L.P.); luciana.rossi@unimi.it (L.R.); 2 Departamento de Producción Animal y Ciencia de los Alimentos, Universidad de Zaragoza-IA2, 50013 Zaragoza, Spain; jennifer.munoz@unizar.es (J.M.-G.); javier.alvarezr@unizar.es (J.A.-R.)

**Keywords:** ammonia, gas production, Pietrain crossbreds, saponins, volatile fatty acids

## Abstract

The effect of *Yucca schidigera* extract (YSE; 10.8% saponins) on in vitro caecal disappearance (IVCD) was tested by incubating pre-digested feed with 0, 150, or 300 mg YSE/kg using caecal inocula from male and female pigs (Experiment 1). The apparent total tract digestibility (ATTD) of nutrients and fermentation products were assessed in vivo in 40 crossbred finishing pigs fed with 0 or 300 mg YSE/kg for 42 days (Experiment 2). In Experiment 1, YSE did not affect IVCD or gas production, but reduced caecal ammonia-N across sexes (*p* < 0.05). Caecal pH decreased progressively with increasing YSE in female-derived inocula only (*p* = 0.015), and volatile fatty acid (VFA) were suppressed in females at the highest dose (*p* = 0.013), while male-derived inocula remained unaffected. Entire males exhibited higher IVCD (*p* < 0.001) and lower ammonia-N (*p* = 0.034) and VFA production (*p* < 0.10) than females. In Experiment 2, YSE did not influence faecal ammonia-N, or VFA profile (*p* > 0.10), but reduced the ATTD of crude protein (*p* < 0.001) and organic matter (*p* < 0.001) relative to the control diet. YSE selectively modulated in vitro caecal fermentation in pigs, but these effects were not confirmed in vivo.

## Simple Summary

To reduce nitrogen losses and ammonia emissions in finishing pigs from Pietrain sires, this integrated in vitro and in vivo approach (Experiment 1 and Experiment 2, respectively) evaluated the effect of dietary *Yucca schidigera* extract (YSE) on nutrient utilization and hindgut microbial fermentation products, with the possibility of sex-dependent responses. YSE selectively modulated in vitro caecal fermentation, with female-derived microbial communities showing greater proteolytic activity and higher susceptibility to saponin-mediated suppression of fermentative output compared with male-derived inocula; however, these effects were not confirmed in vivo. The practical contribution of YSE to reducing ammonia emissions in finishing pigs is therefore more likely mediated through systemic modulation of nitrogen catabolism rather than through direct suppression of hindgut fermentation.

## 1. Introduction

The growing global demand for animal-derived protein has driven the expansion of intensive pig production systems, particularly across Europe. While this intensification has improved production efficiency, it has also increased environmental concerns associated with nutrient losses and gaseous emissions from pig farming. In response, the European Union has progressively implemented stricter regulations aimed at promoting more sustainable livestock systems that reconcile environmental protection, economic viability, and social acceptance [1]. These policies often restrict herd size and limit farm expansion, especially in regions with high livestock density, which may compromise economies of scale and threaten the long-term sustainability of pig production systems [1].

The swine sector is estimated to contribute up to 9% of total greenhouse gas emissions from the global livestock industry, with manure management accounting for approximately 20% of emissions associated with pig production systems [2]. Emissions from pig farms consist mainly of nitrogenous compounds, particularly ammonia (NH_3_), followed by smaller amounts of methane (CH_4_), phosphorus compounds, and various odorous substances. Ammonia emissions are of particular concern because they contribute to atmospheric particulate matter formation, nitrogen deposition, and environmental eutrophication, while also posing risks to human health. In addition, odorous emissions including volatile fatty acids (VFA), sulphur-containing compounds, phenols, indoles, and other nitrogenous gases negatively affect air quality and reduce the social acceptance of pig farming, particularly in peri-urban and densely populated areas [3,4].

Many of these emissions originate from microbial fermentation of undigested dietary components and endogenous substrates in the gastrointestinal tract, particularly in the hindgut. Volatile fatty acids with carbon chain lengths ranging from C2 to C9 contribute substantially to odour formation from manure, while acetate, the most abundant VFA, can serve as a precursor for methane production during microbial fermentation [5,6]. Consequently, nutritional strategies aimed at improving nutrient digestibility and modulating gastrointestinal microbial activity have attracted considerable attention as potential tools to reduce nutrient losses and mitigate gaseous emissions from pig production systems.

Among these approaches, plant-derived feed additives have gained increasing interest due to their natural origin and multifunctional biological properties, including antimicrobial, antioxidant, anti-inflammatory, and surface-active effects that may beneficially modulate gut microbiota composition and fermentation activity [7,8]. *Yucca schidigera*, a desert plant naturally rich in steroidal saponins and polyphenolic compounds, has been widely studied as a feed additive in livestock production. Dietary supplementation with *Yucca schidigera* at low-to-moderate levels (50–200 mg/kg feed) has been shown to modulate caecal fermentation in pigs *in vitro* [7] and *in vivo* [8], as evidenced by increased concentrations of VFA, a shift in the microbiota towards beneficial species such as *Bifidobacterium, Lactobacillus*, and *Bacillus,* and a reduction in pathogenic bacteria like *Escherichia coli*. Such microbial modulation may contribute to improved nutrient utilization and reduced formation of harmful nitrogenous metabolites, including ammonia nitrogen (NH_3_-N).

The reduction in ammonia emissions associated with YSE supplementation has been primarily attributed to the biological activity of steroidal saponins. These compounds possess surface-active properties capable of disrupting microbial cell membranes and modifying microbial fermentation pathways. In addition, the glycosidic moiety of saponins may interact directly with ammonia, thereby limiting its formation and volatilization [9].

In addition to dietary factors, biological differences between sexes may influence nutrient utilization and microbial fermentation in pigs. Sex affects several physiological and metabolic processes, including feed intake, growth performance, body composition, and nitrogen metabolism. These differences may lead to variation in nutrient digestibility and hindgut microbial fermentation patterns between males and females [10]. Therefore, considering sex as a biological factor may provide additional insights into nutritional strategies aimed at improving nutrient efficiency and reducing environmental emissions in pig production.

It was hypothesized that YSE supplementation would improve nutrient utilization and modulate hindgut microbial fermentation in pigs, thereby reducing nitrogen losses and ammonia emissions, with possible sex-dependent differences due to physiological variation in microbial activity and nitrogen metabolism. The present study therefore aimed to evaluate the effects of dietary supplementation with a commercial *Yucca schidigera* extract (YSE) on microbial fermentation products and nutrient utilization in finishing pigs. First, an in vitro dose–response caecal fermentation trial in pigs was conducted to assess the effects of different inclusion levels of YSE on fermentation parameters. Based on these results and practical supplementation recommendations, a subsequent in vivo experiment in pigs was performed to evaluate the effects of including 300 mg YSE/kg feed (as-fed basis) on nutrient digestibility as well as ammonia and VFA emissions in finishing pigs. In addition, the study examined the potential influence of sex on fermentation patterns and nitrogen metabolism. It was hypothesized that YSE supplementation would improve nutrient utilization and modulate hindgut microbial fermentation, thereby reducing nitrogen losses and ammonia emissions, with possible differences between sexes due to physiological variation in microbial activity and nitrogen metabolism.

## 2. Materials and Methods

### 2.1. Feeds and Additives

The additive evaluated in the present study was a commercial extract of *Yucca schidigera* (YSE) (Yucca-50; Anagalide S.A., Barbastro, Huesca, Spain) with a concentration of 50 °Brix (measured at 20 °C), containing 10.8% saponins and an ammonium-binding activity of 117 mg NH_3_/100 mL, which is a measure of the capacity of the extract to bind and neutralise ammonia through interaction between the glycosidic moiety of saponins and NH_3_ molecules, thereby limiting its formation and volatilisation [11,12]. The extract was processed by the manufacturer through maceration in water followed by pressing of the stems. To avoid microbial spoilage, potassium sorbate (E-202) was added in the extract until the mixture achieved a pH of 3.9 ± 0.3.

Two complementary experiments were conducted. The *in vitro* trial (Experiment 1) evaluated dose–response increasing concentrations of YSE (0, 150 and 300 mg of YSE/kg of feed), whereas the in vivo experiment (Experiment 2) evaluated dietary supplementation with 0 or 300 mg of YSE/kg of feed.

The basal diet used in the present study was the same as that described in detail by Santoru et al. [13] (Table 1). Briefly, the diet was formulated to meet the nutrient requirements of finishing pigs weighing between 70 and 120 kg according to FEDNA (2013) (Fundación Española para el Desarrollo de la Nutrición Animal) guidelines [14], with 2527 kcal of Net Energy/kg, 160 g of crude protein (CP)/kg, and 9 g of total Lysine/kg. This diet was selected as it represents a standard finishing diet for lean-genotype pigs under commercial conditions in Spain, ensuring the practical relevance of the results. Feed was offered ad libitum with continuous access to water throughout the experimental period. The chemical analyses in feed were organic matter (OM) (ref. 942.05), CP (ref. 976.05), ether extract (EE) (ref. 2003.05), and NDF using an Ankom 200 Fiber Analyzer (Ankom Technology, New York, NY, USA), using α-amylase and sodium sulphite, with results expressed exclusive of residual ashes [15,16].

### 2.2. Experiment 1: In Vitro Nutrient Disappearance and Caecal Fermentation 

Prior to in vitro cecal fermentation, the experimental diet supplemented with increasing doses of YSE (0, 150 and 300 mg/kg; as-fed basis) was subjected to a pre-digestion procedure in order to simulate the substrate reaching the hindgut. A two-step enzymatic digestion was applied to mimic gastric and small intestinal digestion [17]. During the gastric phase, 1 g of each sample was incubated for 2 h at 39 °C and pH 2.0 with porcine pepsin (≥250 units/mg solid, 10 mg/mL; P7000, Sigma-Aldrich, St. Louis, MO, USA) in 250 mL Erlenmeyer flasks, in a shaking water bath (Unitronic 320-OR, Selecta, Barcelona, Spain). To avoid bacterial fermentation, 0.5 mL of chloramphenicol solution (C0378, chloramphenicol, Sigma-Aldrich, St. Louis, MO, USA) was also added at 5 g/L ethanol. The flasks were closed with a silicon stopper and incubated in a shaking at 39 °C for 2 h. After the incubation, the second step simulated the digestion in the small intestine of pigs. Firstly, 10 mL of phosphate-buffered solution (0.2 M, pH 6.8) and 5 mL of 0.6 M NaOH solution were added in the flasks. Then, the pH was adjusted to 6.8 using 1 M HCl or NaOH solution, and 1 mL of freshly prepared pancreatin solution (50 mg/mL; 4× USP, P1750, pancreatin from porcine pancreas, Sigma-Aldrich, St. Louis, MO, USA) was added. Then, the flasks were incubated in the shaking incubator at 39 °C for 4 h and pH 6.8 with porcine pancreatin (100 mg/mL; P7545, Sigma-Aldrich, St. Louis, MO, USA) in the same shaking water bath. After enzymatic hydrolysis, undigested residues were recovered by filtration (12 h) through ash-free paper filters (pore diameter: 20–25 µm) and dried at 60 °C for 48 h in a forced-air drying oven (Selecta, Barcelona, Spain). The resulting residues were used as substrates for the subsequent in vitro batch fermentation.

The inclusion levels of YSE (150 and 300 mg/kg) were established to simulate a dose–response of the effects of YSE on in vitro gas production (GP) and fermentation pattern of the expected luminal concentrations of the feed additive reaching the hindgut of fattening pigs (70–120 kg body weight).

Cecal inoculum was individually obtained from 2 entire males and 2 female pigs from Pietrain sires (PIC 408, halothane-free) crossed with PIC Landrace × Large White dams, each with a body weight (BW) of 115 kg. All four donor animals received the basal diet without YSE supplementation, offered ad libitum with continuous access to water, as described in Section 2.1 and previously reported in detail by Santoru et al. [13]. Animals were slaughtered at a commercial abattoir (Grupo Arcoiris S.L., Valderrobres, Teruel, Spain) after a 12 h fasting period and 2 h of lairage. Caecal contents were collected immediately after evisceration and transported to the laboratory in hermetic containers containing frozen carbon dioxide, which provided an inert atmosphere and maintained anaerobic and low-temperature conditions during transport. All handling tools and containers were sterilised to prevent external contamination, and samples were clearly labelled according to pig sex and replicate. Upon arrival, the inoculum was gradually warmed from sub-zero temperature to 38 °C in a controlled water bath to restore microbial activity before incubation. The caecal contents from each pig were diluted in a 1:2 ratio with anaerobic phosphate-buffered saline solution (PBS; 0.1 M, pH 7.0; flushed with CO_2_) to achieve an incubation pH of 6.5, in order to mimic the caecal conditions of finishing pigs. The final incubation medium contained 17.5% caecal content and 82.5% buffered medium. The buffered medium was composed of bicarbonate buffer (NaHCO_3_), mineral macro- and microelement solutions, a reducing agent (cysteine-HCl), and a resazurin solution as a redox indicator, prepared as described by Amanzougarene and Fondevila [18]. The incubation solution was dispensed anaerobically into 35 mL Wheaton bottles (Sigma-Aldrich, St. Louis, MO, USA), sealed with butyl rubber stoppers and aluminium crimp caps, with 25 mL of incubation medium per bottle, yielding a headspace volume of approximately 10 mL. Each bottle contained 250 mg of pre-digested substrate and the corresponding dose of YSE.

The feed additive was incorporated directly into the pre-digested substrate prior to fermentation. The experimental design included three Wheaton bottles per dose (0, 150 and 300 mg/kg) and per inoculum donor (with a total of four cecal inoculum from finishing pigs), resulting in 36 incubation bottles in total (3 replicates × 3 treatments × 4 inoculum donors). In addition, two blank bottles per inoculum donor (8 blanks in total) were included. These blank bottles were used to correct for background fermentation activity and contained the buffer and the inoculum, but had no additive or substrate. Incubations were conducted for 10 h at 38 °C, and the GP in the headspace was measured at 2, 4, 6, 8, and 10 h and further released. After each measurement, accumulated gas was released. Pressure was recorded with a HD 2124.02 manometer fitted with a TP804 pressure gauge (Delta Ohm, Caselle di Selvazzano, Italy). Pressure readings were converted to gas volume (mL) using a previously developed calibration equation (detailed in Section 2.4.), established from regression of known gas volumes against measured pressure values, with corrections applied for atmospheric pressure. GP for each time interval was subsequently computed. After 10 h of incubation, bottles were opened and pH was immediately recorded using a calibrated portable pH meter (Seven2Go S2, Mettler Toledo, Columbus, OH, USA). Two subsamples of 1 mL each were then collected from each incubation bottle. One subsample was mixed with 0.5 mL of a deproteinizing solution (0.5 M, H_3_PO_4_) containing 4-methyl valeric acid as the internal standard (2 g/L) for volatile fatty acid (VFA) analysis by gas chromatography in an Agilent 6890 instrument equipped with an FID detector and a capillary column (HP-FFAP Polyethylene glycol TPA, 30 m × 530 μm id). Samples were injected into the column with Helium as the carrier gas and a temperature ramp from 80 to 230 °C. The other sample was acidified with 0.5 mL of HCl (3 M) for NH_3_-N determination. The concentration of ammonia-N was determined spectrophotometrically following the procedure by Chaney and Marbach [19]. Both samples (VFA and ammonia-N) were stored at −20 °C until analysis. Duplicate analysis was carried out in each sample.

Fermentation residues were collected by filtration through filters with a pore diameter of 20–25 µm and dried at 65 °C for 48 h to determine in vitro caecal disappearance (IVCD). The IVCD was calculated as:

$$IVCD\left(\%\right)=\frac{\left[DMfeed-\left(DMresidue-DMblank\right)\right]}{DMfeed}\times 100$$

where DM_feed_(g) is the amount of feed dry matter (DM) incubated, DM_residue_ (g) is the dry matter residue after in vitro digestion and fermentation, and DM_blank_ (g) is the dry matter residue recovered from blank incubations.

### 2.3. Experiment 2: In Vivo Total Tract Digestion, Ammonia and Odour Compounds

#### 2.3.1. Animals and Experimental Design 

Experiment 2 was carried out over 42 days in the facilities of the Animal Experimentation Service of the University of Zaragoza. Crossbred finishing pigs (n = 40; 20 entire males and 20 females), weighing 76.1 ± 5.20 kg at 20–21 weeks of age, were used in the present study. Animals were progeny of Pietrain sires (PIC 408, halothane-free) mated to PIC Landrace × Large White dams. Pigs were allotted to pens (two pigs per pen) on the basis of sex and initial body weight, and pens were randomly assigned to one of two dietary treatments following a randomised complete block design. The experimental diets consisted of a basal diet without additives used as control or the same basal diet supplemented with 300 mg/kg feed of *Yucca schidigera* extract (YSE), pre-mixed with sodium chloride on a weight basis prior to dietary inclusion, as described in Section 2.1. The diet composition and nutrient levels are presented in Table 1 and Section 2.1. The supplementation level was selected based on the dose–response screening performed in vitro caecal fermentation study (Experiment 1), where the highest practical inclusion level induced measurable changes in fermentation traits and was therefore chosen for in vivo evaluation. Pigs were housed in 20 identical pens on fully slatted floors, providing 2 m^2^ per pig, and each pen was equipped with a cup drinker and a vertical feeder. Feed and water were offered ad libitum throughout the 42-day experimental period. Environmental and management conditions were maintained homogeneous across pens as previously described by Santoru et al. [13].

#### 2.3.2. Samples Analysis 

Faecal samples were collected directly from the rectal ampulla of each pig on days 0 (D0), 21 (D21), and 42 (D42) of the experimental period, collecting approximately 150–200 g of fresh faeces per pen, to determine apparent total tract digestibility (ATTD) of nutrients. Samples obtained from the two pigs within each pen were pooled to obtain one representative sample per pen and were stored at −20 °C until analysis. Fresh faecal samples collected on day 42 were also used for the determination of pH, NH_3_-N and VFA concentrations.

Faecal dry matter (DM) content was measured as an indicator of faecal consistency [20]. After drying, samples were ground through a 1 mm screen and analysed in duplicate for organic matter (OM) and crude protein (CP) according to the AOAC procedures previously described [16]. Acid-insoluble ash (AIA) concentration in feed and faeces was determined following the method described previously [21].

Apparent total tract digestibility (ATTD) of OM and CP was estimated using AIA as an internal marker and calculated according to the marker ratio method as follows:

$$ATTD\;of\;OM\;and\;CP\;\left(\%\right)=100\times \left(1-\frac{AIA\;in\;feed}{AIA\;in\;faeces}\times \frac{nutrient\;in\;faeces}{nutrient\;in\;feed}\right)$$

where AIA in feed and AIA in faeces represent the concentrations of acid-insoluble ash in feed and faeces, respectively, and Nutrient in feed and Nutrient in faeces correspond to the concentrations of OM or CP in feed and faeces. The recovery of AIA in faeces was assumed to be complete, as this marker is considered inert and homogeneously distributed in feed and digesta [22].

Faecal pH was measured using a calibrated portable pH meter (Seven2Go S2, Mettler Toledo, Columbus, OH, USA) equipped with a standard glass electrode. For NH_3_-N determination, 2 g of faeces were homogenised in 4 mL of 0.2 N HCl and the extract was subsequently analysed by colorimetric quantification according to the procedure described previously [19]. VFA concentrations were determined by gas chromatography. For this purpose, 1 g of faeces was homogenised in 4 mL of a deproteinising solution containing H_3_PO_4_ (0.5 M) with 4-methyl valeric acid as the internal standard (2 g/L), consistent with the preparation method used for the in vitro VFA samples described in Section 2.2. Samples were analysed using an Agilent 6890 Series gas chromatograph (Agilent Technologies, Santa Clara, CA, USA) equipped with a flame ionisation detector and a HP-FFAP Polyethylene glycol TPA capillary column (30 m × 530 µm i.d.). Injection was performed on-column, using helium as carrier gas (12 mL/min) with a temperature ramp from 80 to 165 to 230 °C. Make-up gas for the detector was helium (25 mL/min). The split ratio was 20:1, and the injector and detector temperatures were set at 200 and 350 °C, respectively. Chromatographic data were processed using HP ChemStation software (version A.08.03; Agilent Technologies, Santa Clara, CA, USA). For NH_3_-N determination, 2 g of faeces were homogenised in 4 mL of 0.2 N HCl and the extract was subsequently analysed by colorimetric quantification according to the method described by Chaney and Marbach [19].

### 2.4. Calculations and Statistical Analyses

Pressure readings were converted to gas volume (mL) using the calibration regression equation y = 1.7033x + 0.272 (R^2^ = 0.98), established from regression of known gas volumes against measured pressure values, with corrections applied for atmospheric pressure, following the procedure described by Amanzougarene and Fondevila [18]. Given the short incubation period (10 h) used to simulate caecal fermentation conditions in pigs, the average rate of gas production (mL/h) at each measurement interval (0–2, 2–4, 4–6, 6–8, and 8–10 h) was considered as the difference in cumulative GP between consecutive time points, and the total cumulative GP over 10 h was used as the primary fermentation endpoint.

All statistical analyses were performed using RStudio software (R version 4.4.0, R Core Team, 2024). Data from the in vitro fermentation trial (Experiment 1) were analysed to evaluate the dose–response effects of *Yucca schidigera* extract (YSE) using mixed linear models. The experimental unit was the incubation bottle. The statistical model applied was:

Y_ijklm_ = μ + D_i_ + S_j_ + T_k_ + (DxS)_ij_ + (DxT)_ik_ + *P_l_* + *e_ijklm_*

where Y_ijklm_ is the dependent continuous variable, *μ* is the overall mean, *D_i_* is the fixed effect of the dose (*i* = 0 vs. 300 mg/kg) expressed as Treatment (Trt), *S_j_* is the fixed effect of sex (*j* = male vs. female), T_k_ is the fixed effect of sampling time (*k* = hour 2, 4, 6, 8 or 10 in the in vitro trial or day 21 and 42 in the in vivo trial), (DxS)_ij_ is the interaction between dose and sex (DxT)_ik_ is the interaction between dose and time, *P_l_* is the random effect of the pig inoculum (j = 1 to 4 in the in vitro trial) or the pen (l = 1 to 20 in the in vivo trial) and *e_ijklm_* is the residual error. The random effect accounted for the correlation among repeated observations and corrected for potential differences in baseline values among individuals. Only the significant interactions (*p* < 0.05) are reported in Section 3.

For ATTD of CP and OM variables, faecal DM content was included in the model as a covariate. When significant treatment effects were detected, means were compared using Tukey’s multiple comparison test. Statistical significance was declared at *p* < 0.05, whereas values of *p* between 0.05 and 0.10 were considered indicative of a tendency. 

## 3. Results

### 3.1. Experiment 1: Effect of Dietary Yucca and Sex on In Vitro Caecal Fermentation

To evaluate the effects of dietary supplementation with YSE on in vitro caecal fermentation, caecal inocula from male and female donors were incubated with YSE (Table 2, Table 3 and Table 4). Under the experimental conditions, YSE supplementation did not significantly affect IVCD (*p* > 0.10). However, a significant effect of inoculum sex was observed, with higher IVCD values recorded in males than in females across all diets (*p* < 0.001). No significant Trt × Sex interaction was detected for IVCD (*p* = 0.983).

In contrast, caecal pH was significantly influenced by diet (*p* = 0.007), and a Trt × inoculum sex interaction was observed (*p* = 0.015). In male inocula, pH tended to decrease with increasing YSE, with YSE150 and YSE300 differing significantly from the control (*p* < 0.05), while female pH remained stable across all dietary treatments.

Dietary supplementation with increasing dose of YSE did not affect in vitro gas production (Table 3) at any incubation time nor total GP (*p* > 0.10). In contrast, inoculum sex significantly influenced GP throughout the incubation period, with higher GP values recorded in bottles inoculated with female caecal inoculum compared with male inoculum (*p* < 0.001). Peak GP was reached earlier in female inocula (4 h) than in male inocula (6 h). A significant Trt × inoculum sex interaction was detected at 2 h of incubation (*p* = 0.011), indicating that YSE supplementation reduced GP in male inocula at the control and YSE150 diets, whereas no clear dietary effect was observed in female inocula at this time point. No interactions between diet and inoculum sex were observed at later incubation times or for total GP (*p* > 0.10).

The *in vitro* evaluation of ammonia-N and VFA concentrations after 10 h of fermentation (Table 4) showed that YSE significantly affected both parameters. Dietary supplementation with YSE reduced ammonia production (*p* < 0.05), and a significant effect of inoculum sex was also observed (*p* = 0.034), with higher ammonia concentrations recorded in female than in male inocula. However, the Trt × inoculum sex interaction was not significant (*p* > 0.10). Regarding VFA production expressed per unit of degraded dry matter, a significant Trt × inoculum sex interaction was detected for total VFA concentration (*p* = 0.013). In female inocula, total VFA concentration was similar in control and YSE150, whereas it decreased in YSE300 compared with control values. In male inocula, total VFA concentration remained stable across all dietary treatments. Overall, females tended to produce higher total VFA concentrations than males (*p* < 0.10).

Significant Trt × inoculum sex interactions were also observed for all individual VFA analysed (*p* < 0.05). Absolute concentrations of acetate, propionate, isobutyrate, butyrate, isovalerate and valerate were modified by dietary treatment in female inocula, showing non-linear response with similar values in YSE150 and control and reduced values in YSE300 compared to the control, while remaining relatively constant in male inocula. In contrast, no significant effects of Trt, inoculum sex or their interaction were detected for the relative molar proportions of VFA (*p* > 0.10).

### 3.2. Experiment 2: In Vivo Chemical Composition of Faeces and Nutrient Digestibility 

To evaluate the effects of dietary supplementation with YSE on faecal characteristics and nutrient digestibility, an in vivo trial lasting 42 days was conducted in pigs (Table 5). Faecal DM content was not significantly influenced by Trt (*p* > 0.10), but increased over time (*p* < 0.001). Sex effect was observed, with females showing higher faecal DM than males throughout the experimental period (*p* = 0.006), and the highest difference was observed at day 42.

Faecal pH was not modified by dietary treatment or sex (*p* > 0.10), with mean values of 6.54 and 6.73 (±0.203) for the control and YSE treatments, respectively, and 6.67 and 6.60 (±0.203) for entire males and females.

The concentration of acid-insoluble ash (AIA) in faeces concentration was markedly influenced by Trt (*p* < 0.001), time (*p* < 0.001) and their interaction (*p* < 0.001). The AIA increased in pigs fed with control, whereas pigs receiving YSE showed lower AIA concentrations during the experimental period.

Faecal CP concentration decreased with time (*p* < 0.001) but was not affected by Trt or sex (*p* > 0.10). Apparent total tract digestibility (ATTD) of CP was influenced by Trt (*p* < 0.001), time (*p* < 0.001) and their interaction (*p* < 0.05), with pigs fed the YSE diet showing lower CP digestibility that remained relatively stable over time. No sex effect was detected for CP digestibility (*p* > 0.10).

Similarly, ATTD of OM was affected by Trt (*p* < 0.001), time (*p* < 0.01) and their interaction (*p* < 0.001). OM digestibility increased over time in pigs fed the control, whereas it remained lower and relatively stable in pigs receiving YSE. No differences between sexes were observed (*p* > 0.10).

### 3.3. Experiment 2: In Vivo Ammonia and VFA Content in Faeces

Under the experimental conditions of the in vivo trial, dietary supplementation with YSE at 300 mg/kg had limited effects on faecal fermentation metabolites at day 42 (Table 6). Faecal ammonia concentration was not influenced by dietary treatment, sex or their interaction (*p* > 0.10). Likewise, total VFA concentration and the absolute concentrations of individual VFA were not modified by YSE supplementation (*p* > 0.10). Similarly, the relative molar proportions of VFA were not affected by Trt or sex (*p* > 0.10).

## 4. Discussion

### 4.1. Effect of Yucca Schidigera on In Vitro Cecal Fermentation

An in vitro study was conducted to evaluate the potential effects and mode of action of *Yucca schidigera* supplementation on hindgut fermentation and dry matter disappearance (IVCD) in finishing pigs across three inclusion levels (0, 150, and 300 mg/kg) (Experiment 1). Caecal inocula were obtained from finishing pigs fed a diet composed mainly of cereals (maize, wheat, and barley) and soybean meal, which is representative of standard commercial finishing conditions in Spain. As expected, the inocula had low pH (5.95 ± 0.182) across treatments and high gas production (1525 ± 439 ml/g degraded DM) similar to those previously reported in the caecum of finishing pigs fed cereal-soybean meal-based diets (13.5–14.5% CP), where caecal pH ranged from 5.65 to 6.06 and 1200–1600 mL gas volume/g DM [23,24]. Notably, most of the characteristics observed in the inoculum persisted after 10 h of incubation, indicating that the in vitro method successfully mimicked the in vivo conditions. However, the cecum microbiota was not investigated in this study, limiting the ability to assess potential decreases in specific microbial populations, particularly those that are not readily culturable in vitro. These preliminary findings were subsequently validated through an in vivo trial in finishing pigs, as described in Experiment 2.

Steroidal saponins present in YSE have been shown to enhance animal productivity mainly due to their potent antimicrobial activity in the hindgut [25]. Specifically, dietary supplementation with YSE at inclusion levels ranging from 100 to 200 mg/kg, providing approximately 10–20 mg saponins/kg feed, has been associated with selective inhibition of urease-producing bacteria [26]. At a dietary inclusion of 120 mg/kg, YSE has been shown to adsorb to the microbiota and solids of the porcine caecum, selectively inhibiting urease-producing bacteria at low cell densities, thereby reducing hindgut protein degradation and ammonia production [26]. Consistent with this mechanism, Fan et al. [12] reported that YSE supplementation at 120 mg/kg significantly decreased hindgut NH_3_-N production and regulated VFA composition in the distal intestine of weaned piglets, effects associated with improved nutrient digestibility and gut barrier function [12]. Under our experimental conditions, YSE supplementation maintained caecal IVCD across all inclusion levels, suggesting that YSE did not impair substrate degradability by the caecal microbiota, and that the antimicrobial activity of steroidal saponins was selective rather than broadly inhibitory towards fermentative bacteria. This finding is consistent with Anele et al. [27],who reported no effect of a YSE-based feed additive included at 500 mg/kg on in vitro dry matter disappearance across multiple substrate types including maize, soybean meal and mixed diets, at incubation times of 24 and 48 h in ruminants. In contrast, in vivo studies in pigs have reported improved apparent DM and nutrient digestibility following YSE supplementation at 120–300 mg/kg [12,28], suggesting that the benefits of YSE on nutrient utilization may be primarily mediated through improvements in small intestinal morphology, digestive enzyme activity, and mucosal barrier function [12], rather than through enhanced hindgut fermentative degradation.

Regarding caecal pH, YSE supplementation induced a linear decrease across inclusion levels, being most pronounced at the highest dose (YSE300). This pH reduction is consistent with findings by Yang et al. [8], who reported that dietary YSE significantly decreased intestinal pH in weaned piglets, an effect attributed to selective inhibition of urease-producing and deaminating bacteria. The reduction in pH is indicative of increased acidic fermentation, which could promote the growth of beneficial acid-tolerant microorganisms such as lactobacilli, while simultaneously reducing ammonia accumulation in the hindgut [12]. Despite this pH reduction, total gas production was not affected by YSE at any inclusion level, either cumulatively or at individual incubation time points between 4 and 10 h. The consistency between unchanged gas production and stable IVCD values across treatments collectively confirms that YSE exerted a targeted antimicrobial effect on proteolytic populations without broadly disrupting caecal fermentative activity. Beyond its antimicrobial properties, steroidal saponins present in YSE are also known to interact with sterol moieties in intestinal mucosa cell membranes, potentially facilitating nutrient absorption and improving animal performance. The magnitude of these effects is, however, highly dependent on the botanical source, inclusion level, dietary composition, and the specific physiological conditions of the animals [12].

The consistent decrease in caecal ammonia-N concentration observed across YSE inclusion levels, maintained alongside stable branched-chain VFA concentrations, warrants further mechanistic consideration. Given that iso-butyrate and iso-valerate originate from the microbial degradation of valine and leucine, respectively, the maintenance of these compounds implies that protein fermentation pathways remained largely intact, and that ammonia-N availability was sufficient to sustain microbial metabolic activity [29]. The concurrent maintenance of total VFA output alongside lower ammonia-N concentrations, however, points to an alternative explanation: that a higher proportion of dietary nitrogen may have been assimilated into microbial biomass rather than released as free ammonia, potentially reflecting an upregulation of microbial protein synthesis [12]. Another hypothesis is the selective inhibitory effect of saponins on hyper-ammonia-producing bacterial populations [25]. This interpretation is supported by Fan et al. [12], who demonstrated that YSE supplementation at 120 mg/kg in weaned piglets decreased hindgut ammonia-N production in association with enhanced nutrient utilization and improved gut barrier integrity, suggesting that reduced luminal ammonia may reflect more efficient nitrogen retention at the intestinal level rather than a suppression of fermentative activity per se. Similarly, previous in vitro studies reported that YSE supplementation at 150–300 mg/kg in pig faecal slurries from growing pigs reduced ammonia formation by 30–50% after 36–48 h incubation while preserving VFA production, linking reduced NH_3_ to improved pig health and lower housing emissions [7].

### 4.2. Effect of Yucca Schidigera on In Vivo Cecal Fermentation

To validate the in vitro findings, an in vivo experiment (Experiment 2) was performed to assess the effect of YSE supplementation at 300 mg/kg feed on nutrient digestibility and hindgut fermentation metabolites over 42 days. The *Yucca schidigera* extracts rich in steroidal saponins and glycocomponents have traditionally been associated with increased apparent total tract digestibility (ATTD) of organic matter (OM), dry matter (DM), and crude protein (CP) in pigs [30], however, our in vivo results revealed a more complex pattern of responses. The dose selected was 300 mg/kg of extract per kg of feed, as it was the dose which most consistently influenced the parameters evaluated in the in vitro trial.

The progressive increase in faecal DM content observed throughout the 42-day experimental period is consistent with the physiological maturation of the gastrointestinal tract as pigs approach market weight. As the hindgut matures, the colon exhibits an enhanced capacity for water and electrolyte reabsorption, driven by increased digesta retention time and the establishment of a more metabolically active microbial community [31]. The production of VFAs by hindgut microbiota stimulates sodium-coupled water absorption across the colonic epithelium, contributing to the progressive consolidation of faecal matter over time. Notably, faecal DM was not influenced by dietary treatment throughout the experimental period, suggesting that YSE supplementation at 300 mg/kg did not substantially alter colonic water absorption dynamics or broadly disrupt the fermentative activity of the hindgut microbiota. This is consistent with the *in vitro* findings from Experiment 1, where IVCD and total gas production remained unaffected across YSE inclusion levels, collectively confirming that steroidal saponins at this dose exerted a selective rather than broadly inhibitory antimicrobial effect.

Faecal pH was likewise unaffected by dietary YSE inclusion, with mean values of 6.54 and 6.73 (±0.203) for the control and YSE treatments, respectively. This contrasts with the significant linear decrease in caecal pH observed in vitro with increasing YSE inclusion, and with previous in vivo reports in weaned piglets where dietary YSE reduced intestinal pH through selective inhibition of urease-producing and deaminating bacteria [12]. The absence of a pH response in finishing pigs may reflect the higher buffering capacity of hindgut digesta in animals fed a concentrate-based diet at this physiological stage, or a reduction in the bioavailability of active saponin fractions along the gastrointestinal tract before reaching the large intestine. The magnitude of YSE effects on hindgut fermentation pH is known to be highly dependent on animal age, dietary composition, and the saponin concentration of the specific extract used [11], which likely accounts for the discrepancy between the in vitro and in vivo responses observed in the present study.

The concentration of AIA in faeces was markedly influenced by diet, time, and their interaction, with different patterns observed between treatments over the experimental period. In control pigs, AIA concentration increased progressively over time, a pattern consistent with improving nutrient digestibility as the digestive system matures, since higher nutrient absorption results in a higher relative concentration of the indigestible marker in faecal residues [32]. In contrast, pigs receiving YSE showed lower and more stable AIA concentrations throughout the experiment. This atypical pattern suggests that YSE supplementation may have altered digesta flow dynamics in the hindgut, potentially through changes in passage rate, microbial biomass output, or water-binding properties of the digesta matrix, rather than reflecting a true reduction in digestive efficiency per se [30]. Since AIA is used as an endogenous marker to calculate ATTD, any treatment-related alteration in marker recovery will directly influence the derived digestibility coefficients, and this caveat should be considered when interpreting the digestibility data presented below.

Faecal CP concentration decreased with time but was not affected by dietary treatment, indicating that despite the lower apparent digestibility calculated for YSE-supplemented pigs, the absolute amount of nitrogen recovered in faeces did not differ between groups. This dissociation between faecal CP concentration and calculated ATTD suggests that the reduction in apparent CP digestibility in YSE pigs may be at least partially attributable to the altered AIA recovery described above, rather than solely to impaired protein digestion. Nevertheless, a contribution of steroidal saponins to reduced CP digestibility through antinutritional mechanisms cannot be excluded. Saponins are known to form stable complexes with dietary proteins, reducing their accessibility to proteolytic enzymes, and to inhibit the activity of chymotrypsin, thereby limiting hydrolysis of peptide bonds in the small intestine [12]. These effects are considered dose-dependent, with detrimental impacts on protein digestibility reported more consistently at higher inclusion levels, whereas moderate doses have been associated with neutral or beneficial outcomes [33].

The ATTD of OM followed a similar pattern to CP, being significantly influenced by diet, time, and their interaction. OM digestibility increased progressively over time in control pigs, again consistent with gastrointestinal maturation across the finishing period, while remaining lower and relatively stable in YSE-supplemented animals. The stable trajectory of both CP and OM digestibility in YSE pigs, contrasting with the progressive improvement observed in controls, suggests that YSE supplementation may have attenuated the normal developmental enhancement of digestive capacity rather than causing an acute reduction in nutrient absorption. This interpretation is consistent with the hypothesis that saponin-protein and saponin-lipid complexes limit the bioavailability of substrates for both enzymatic digestion in the small intestine and fermentative degradation in the hindgut, thereby stabilising overall nutrient utilisation across the trial period. The inconsistency with studies reporting improved DM, CP, and OM digestibility following YSE supplementation [12,34] likely reflects differences in animal age, physiological stage, and the saponin concentration of the extracts used, as the beneficial effects of YSE on digestive morphology and enzyme activity appear most pronounced in younger pigs with a less established gastrointestinal microenvironment.

Ammonia in pig housing is predominantly generated not through direct faecal proteolytic fermentation, but through the enzymatic hydrolysis of urinary urea by urease-producing bacteria present in faeces once urine and faeces come into contact within the slurry pit [35]. Measuring faecal ammonia-N in isolation therefore captures only a fraction of the total ammonia pool relevant to housing emissions and may not adequately reflect the inhibitory potential of saponin residues excreted in faeces on urease activity at the urine-faeces interface. This mechanistic distinction may explain why several studies have reported reductions in housing ammonia emissions from YSE-supplemented animals in the absence of corresponding changes in faecal ammonia-N concentration [35]. Importantly, systemic evidence of a YSE-mediated effect on nitrogen metabolism was documented in a parallel trial conducted on the same animals [13], where serum urea concentration remained stable in YSE-supplemented pigs throughout the experimental period while increasing significantly in the control group, with the serum urea/creatinine ratio decreasing significantly in the YSE treatment by day 42. These findings suggest that YSE supplementation modulated the metabolic nitrogen pool at the systemic level, potentially by reducing the rate of hepatic urea synthesis or by shifting nitrogen excretion away from urinary pathways. If a lower urinary urea load was excreted by YSE-supplemented pigs, the substrate available for ammonia generation via urease activity at the urine-faeces interface in the slurry would be correspondingly reduced, providing a plausible systemic mechanism for ammonia emission mitigation that would not be detectable through faecal ammonia measurements alone. This interpretation is consistent with the report by Panetta et al. [35], who demonstrated a marked reduction in urinary ammonia levels in YSE-supplemented pigs at 125 mg/kg in the absence of significant changes in faecal ammonia content, supporting the hypothesis of a shift in the nitrogen excretion route rather than a direct suppression of hindgut proteolysis. Moreover, Chen et al. [26] found no effect of YSE on urease activity in sows, suggesting that direct ammonia-binding by saponin glycocomponents, or systemic attenuation of urea production as observed in the present study, may represent more relevant mechanisms at the doses commonly employed in finishing pig production, rather than enzymatic inhibition per se.

The lack of a significant effect of YSE on faecal VFA concentrations and profiles confirmed that YSE did not broadly suppress fermentative activity in the hindgut of finishing pigs at this inclusion level [12]. This is further supported by the unchanged faecal DM and pH reported above, indicating that the overall fermentative environment of the large intestine was not substantially disrupted by YSE supplementation at 300 mg/kg.

Although the literature on the use of YSE to control odour emissions from pig housing is substantial [25], most studies have focused primarily on total gaseous emissions and ammonia release from faecal fractions, leaving the contribution of VFA to manure odour comparatively underexplored. Volatile fatty acids, alongside ammonia, are major determinants of manure odour, with medium- and short-chain compounds such as iso-butyrate, butyrate, iso-valerate, and valerate being particularly associated with malodour formation [6]. In the present study, YSE exerted only a modest effect on total VFA production and profile in vitro, and this response was not reproduced in vivo, as neither total faecal VFA concentration nor individual VFA profiles differed significantly between dietary treatments. Katsunuma et al. [36], using a YSE inclusion level of 50 mg/kg, similarly reported no significant change in total caecal VFA concentration, although a compositional shift towards higher butyrate, propionate, and valerate at the expense of acetate was observed in post-weaning pigs [36]. The absence of a comparable shift in the present study most likely reflects differences in animal age and physiological stage, as finishing pigs (70–120 kg body weight) have a fully established and compositionally resilient hindgut microbial community that is less responsive to the compositional effects of bioactive additives than the immature microbiota of post-weaning animals. The only tendency towards a treatment effect observed in the in vitro model was a dose-dependent reduction in valerate concentration with increasing YSE inclusion. This is mechanistically relevant, as branched-chain and straight-chain VFAs including iso-butyrate, valerate, and iso-valerate are predominantly generated through the microbial catabolism of amino acids during proteolytic fermentation in the hindgut [37]. A selective inhibitory effect of saponins on proteolytic bacterial populations could therefore reduce the substrate available for valerate synthesis, consistent with the concurrent reduction in caecal ammonia-N observed in vitro. The fact that this tendency was not sustained in vivo further supports the view that the hindgut microbiota of finishing pigs is compositionally resilient and less susceptible to saponin-mediated modulation at moderate inclusion levels, where saponin-protein complexes may sequester fermentable substrates from bacterial enzymatic action but are insufficient to substantially alter the overall fermentation of a mature and diverse microbial community [31]. Consistent with previous reports [38], the overall emission-mitigating role of YSE in swine production appears to be determined primarily by its effect on ammonia rather than on other malodorous compounds, and its practical benefit is likely most pronounced under housing conditions where urine and faeces are allowed to mix within the slurry pit.

### 4.3. Effect of Sex on In Vitro Caecal Fermentation

Sex is a well-recognised biological factor influencing multiple physiological and metabolic processes in pigs, including feed intake, growth rate, body composition, and nitrogen metabolism [10]. However, its potential influence on hindgut microbial fermentation patterns has received comparatively little attention, particularly in the context of nutritional strategies aimed at reducing nitrogen losses and environmental emissions. Given that the efficacy of bioactive feed additives such as YSE may depend on the composition and metabolic activity of the resident hindgut microbiota, and that the gut microbiome of pigs is known to be significantly influenced by host sex [39,40], the present study included inocula from both entire male and female donors to examine whether sex constitutes a relevant source of variation in caecal fermentation outcomes. This approach was motivated by the hypothesis that sex-related differences in microbial community structure and nitrogen metabolism could modulate both baseline fermentation parameters and the magnitude of the fermentative response to YSE supplementation, with potential implications for the design of nutritional strategies targeting emission reduction in sex-segregated finishing systems.

The results confirmed this hypothesis, as inoculum donor sex significantly influenced most hindgut fermentation parameters independent of dietary treatment. Entire males showed significantly higher IVCD values than females across all dietary treatments, and no Diet × Sex interaction was detected for this parameter, indicating that the difference in substrate degradation capacity between sexes was consistent regardless of YSE inclusion level. This finding is consistent with evidence reported by Yao et al. [41], who observed higher abundances of fibre-degrading bacterial taxa in male pigs, including *Ruminococcaceae*, *Clostridium*, and the *Christensenellaceae R-7* group, while females harboured higher proportions of Bifidobacterium and produced higher concentrations of propionate. Communities enriched in fibre-degrading bacteria would be expected to achieve higher rates of polysaccharide hydrolysis and DM disappearance under in vitro conditions, providing a plausible microbial basis for the higher IVCD consistently observed in male-derived inocula. Sex-hormone-mediated differences in microbial community composition, potentially operating through differences in bile acid metabolism and intestinal transit time, may further contribute to these divergent fermentation phenotypes [42].

Caecal pH was not significantly affected by inoculum sex as a main effect; however, a significant Diet × Sex interaction was observed, with increasing YSE doses resulting in a progressive reduction in pH specifically in female-derived inocula, while pH remained unchanged across all inclusion levels in male inocula. This differential pH response between sexes is not attributable to differences in baseline fermentation conditions alone, but rather reflects the divergent susceptibility of the two microbial communities to YSE-mediated perturbation. The female-derived microbiota, characterised by a higher abundance of proteolytic and urease-producing populations as evidenced by the higher baseline ammonia-N concentrations discussed below, would present a higher density of susceptible bacterial targets for saponin-mediated inhibition, thereby producing a more pronounced shift towards acidic fermentation end-products upon YSE exposure. In contrast, the compositional resilience of the male-derived microbiota, dominated by fibre-degrading taxa less sensitive to saponin-mediated membrane disruption [11], would be expected to maintain fermentative balance and hence caecal pH regardless of YSE inclusion, which is precisely what was observed.

The gas production kinetics revealed an additional dimension of sex-related fermentation differences. Female inocula reached peak gas production earlier (4 h) than male inocula (6 h), despite producing higher cumulative gas volumes throughout the incubation period. This gas production profile suggests a metabolic trade-off within female-derived microbial communities between fermentation rate and substrate utilisation efficiency, whereby rapid initial fermentation does not translate into proportionally higher dry matter disappearance. Such a discrepancy implies that a higher proportion of fermentative activity in female-derived communities is directed towards the rapid catabolism of readily fermentable soluble fractions, rather than the more sustained degradation of structural components that typically drives dry matter disappearance over longer incubation periods [42]. This pattern of accelerated but less efficient fermentation in females may reflect a microbial community structure orientated towards rapid nutrient turnover, potentially shaped by the host’s distinct hormonal and bile acid profiles, which are known to influence gut redox potential and microbial metabolic niches [39,42].

The significant Diet × Sex interaction detected at 2 h of incubation provides further evidence of sex-specific microbial responses to YSE. At this early fermentation stage, YSE supplementation reduced gas production in male inocula at the control and intermediate inclusion levels, whereas no inhibitory effect was observed in female inocula at the same time point. This transient response in male inocula may reflect an initial sensitivity of the male-derived microbiota to the surfactant properties of steroidal saponins at the onset of fermentation, which can transiently modulate membrane permeability and disrupt early microbial metabolic activity [11].

Female inocula produced significantly higher ammonia-N concentrations than male inocula after 10 h of fermentation, and this sex effect was independent of dietary treatment as no significant Diet × Sex interaction was detected for this parameter. The higher ammonia-N concentrations in female-derived incubations are indicative of a microbial community with a higher capacity for proteolytic catabolism relative to saccharolytic degradation [37], consistent with the lower IVCD observed in female inocula.

Regarding VFA production, females tended to produce higher total VFA concentrations than males, driven by elevated absolute concentrations of all individual VFAs measured, which is likewise consistent with higher overall fermentative activity in female-derived communities. Notably, the relative molar proportions of individual VFA were not significantly affected by sex, suggesting that while the magnitude of fermentation end-product output differs between sexes, the qualitative profile of fermentation pathways is broadly conserved under standard dietary conditions. The significant Diet × Sex interactions observed for total and all individual VFA concentrations further indicate that the female hindgut microbiota was substantially more susceptible to saponin-mediated suppression of fermentative output than the male microbiota. In female-derived inocula, YSE supplementation at the highest inclusion level suppressed the absolute concentrations of all individual VFAs, whereas male-derived inocula maintained stable VFA output across all dietary treatments. This differential response likely reflects the inherent compositional differences between the two microbial communities described above, whereby the higher abundance of proteolytic bacteria in female-derived inocula renders them more susceptible to the membrane-disrupting activity of steroidal saponins [11].

### 4.4. Effect of Sex on In Vivo Faecal Characteristics, Nutrient Digestibility, and Hindgut Fermentation Parameters

In contrast to the pronounced sex-related differences observed in vitro, the influence of sex on most in vivo faecal and digestibility parameters was limited. Faecal DM content was significantly higher in females than in entire males throughout the experimental period, with the greatest divergence observed at day 42. The higher faecal DM in females may reflect a longer colonic retention time in females relative to entire males, which would promote more extensive water reabsorption across the colonic epithelium and result in drier, more consolidated faecal matter. Sex-related differences in gastrointestinal transit time and colonic motility are known to be modulated by sex hormones in pigs [10], and the peri-pubertal animals used in the present study were likely entering a phase of active hormonal change that may have accentuated these physiological differences by the end of the trial. This observation is broadly consistent with the in vitro evidence of higher fermentative activity in female-derived caecal communities, which could contribute to increased VFA production and hence enhanced colonic water absorption in females under in vivo conditions.

Faecal pH, faecal CP concentration, and the ATTD of both CP and OM were not significantly affected by sex, indicating that the overall digestive efficiency of entire males and females was comparable under the conditions of the present study. This finding is consistent with Álvarez-Rodríguez et al. [43], who reported similar nutrient digestibility between entire males and females in a comparable Pietrain-derived lean genetic line and suggests that the sex-related differences in hindgut microbial fermentation phenotype observed in vitro do not translate into measurable divergence in apparent total tract nutrient utilisation under in vivo conditions. The discrepancy between the in vitro and in vivo sex effects on fermentation parameters likely reflects the integrative nature of in vivo digestibility measurements, which capture the combined contribution of both small intestinal enzymatic digestion and hindgut microbial fermentation and may be less sensitive to sex-related differences in hindgut fermentation activity alone than the isolated caecal fermentation model employed in Experiment 1. Furthermore, Verschuren et al. [39] reported higher feed digestibility in females compared to castrated males, while Crocker and Robison [20] observed lower nutrient concentrations in the faeces of females relative to castrated males, attributing this to the higher growth rates in males and consequent increased nutrient turnover [20]. The discrepancies between these findings and the present results are likely attributable to differences in genetic background and castration status, as the modern lean crossbred entire males used in our trial exhibit higher lean tissue deposition rates and nitrogen retention capacity than the castrated males used in earlier studies, potentially narrowing sex-related differences in nutrient utilisation efficiency.

No significant differences were observed between sexes in faecal ammonia-N concentration or in the total and individual VFA concentrations and molar proportions, indicating that sex did not influence the fermentation metabolite profile of faeces under in vivo conditions. These findings align with Graziosi et al. [44], who also reported no sex-related variation in faecal ammonia or VFA profiles in finishing pigs In contrast, Crocker and Robison [20] found higher faecal ammonia levels in castrated males; however, the genetic lines used in that study are characterised by lower lean tissue deposition rates compared to the Pietrain-derived line used here, and consequently lower nitrogen use efficiency, which may account for the higher faecal ammonia excretion observed in males in that context [20].

## 5. Conclusions

This study demonstrated that dietary supplementation with YSE at 300 mg/kg reduced caecal ammonia-N production in vitro while preserving overall fermentative activity. However, this effect was not observed in vivo, where faecal ammonia-N and VFA concentrations remained unaffected. However, YSE supplementation reduced the in vivo apparent total tract digestibility of crude protein and organic matter compared to controls. Additionally, donor sex significantly influenced in vitro caecal fermentation, with female-derived communities showing greater proteolytic activity and higher susceptibility to saponin-mediated effects.

## Figures and Tables

**Table 1:** Ingredient and nutrient composition of the control and experimental diet (g/kg, as-fed basis).

Feed Ingredients	g/kg
Corn	230
Wheat	160
Barley	210
Soybean meal	200
Bakery meal	164
Animal fat	16.2
Calcium carbonate	8.8
Sodium chloride	3.5
Dicalcium phosphate	1.9
L-lysine 50%	2.5
Vitamins, minerals and acidifiers mix	2.0
Enzyme mix (phytase, protease, carbohydrase)	1.1
L-methionine 88%	0.1
**Analysed nutrient composition**	
Dry matter	873.5
Crude protein	160
Ether extract	52.8
Neutral detergent fiber	112.2
Ash	43.7

**Table 2:** Dose–response effects of *Yucca schidigera* extract (YSE) at 0, 150 and 300 mg/kg feed on in vitro caecal dry matter disappearance (IVCD) and fermentation pH using cecal inoculum from male and female pigs (Experiment 1).

	Control		YSE150		YSE300		SEM ^‡^	*p*-Value		
	Male	Female	Male	Female	Male	Female		Trt	Sex	Trt × Sex
IVCD ^†^ (%)	23.8 ^a^ (74.2)	13.4 ^b^ (63.8)	23.8 ^a^ (73.5)	13.3 ^b^ (63.1)	23.8 ^a^ (77.0)	13.4 ^b^ (66.6)	0.197	0.771	<0.001	0.983
pH	5.85 ^ab^	6.12 ^a^	5.78 ^b^	6.12 ^a^	5.75 ^b^	6.12 ^a^	0.252	0.007	0.458	0.015

^†^ IVCD: Values in parentheses represent total dry matter disappearance obtained as the sum of the in vitro two-step enzymatic digestion (stomach and small intestine) and the in vitro cecal fermentation phase. ^‡^ SEM, standard error of the mean (n = 12 for Trt and n = 18 for inoculum sex). ^a,b^ means within a row or column for the same parameter with different superscripts differ significantly according to Trt and/or inoculum sex (*p* < 0.05).

**Table 3:** Dose–response of *Yucca schidigera* extract (YSE) at 0, 150 or 300 mg/kg of feed on in vitro gas production (mL/of degraded dry matter) using cecal inoculum from male and female pigs (Experiment 1).

	Gas Production (mL/g of Degraded Dry Matter) at 2 h Intervals and Total Sum at 10 h					
	2 h	4 h	6 h	8 h	10 h	Total
Control						
Male	76.3 ^y^	227	393	286	189	1171
Female	259 ^x^	597	484	359	288	1988
YSE150						
Male	35.7 ^z^	203	360	280	189	1068
Female	274 ^x^	567	466	340	274	1922
YSE300						
Male	77.5 ^yz^	231	360	286	190	1144
Female	205 ^xy^	573	474	336	272	1860
SEM ^†^	17.1	65.0	26.8	63.9	52.0	92.7
*p*-value						
Trt	0.312	0.720	0.332	0.714	0.738	0.246
Sex	<0.001	<0.001	<0.001	<0.001	<0.001	<0.001
Trt × Sex	0.011	0.904	0.813	0.808	0.684	0.431

^†^ SEM, standard error of the mean (n = 12 for Trt and n = 18 for inoculum sex). ^x–z^ Means within a column with different superscripts differ significantly according to diets and/or sexes at each incubation time (*p* < 0.05).

**Table 4:** Dose–response of *Yucca schidigera* extract (YSE) at 0, 150 or 300 mg/kg of feed on in vitro ammonia-N and volatile fatty acid (VFA) concentrations after 10 h of caecal fermentation using cecal inoculum from male and female pigs (Experiment 1).

	Control		YSE150		YSE300		SEM ^†^	*p*-Value		
	Male	Female	Male	Female	Male	Female		Trt	Sex	Trt × Sex
Ammonia (µmol/g of degraded dry matter)	170 ^c^	524 ^a^	169 ^c^	486 ^ab^	157 ^c^	470 ^b^	45.3	0.033	0.034	0.196
VFA content (mmol/g of degraded dry matter)										
Total	1.28 ^b^	2.04 ^ab^	1.27 ^b^	2.29 ^a^	1.35 ^b^	1.90 ^b^	0.195	0.117	0.097	0.013
Acetate	0.613 ^ab^	0.907 ^ab^	0.610 ^ab^	1.02 ^a^	0.643 ^ab^	0.843 ^b^	0.116	0.104	0.198	0.016
Propionate	0.136 ^b^	0.259 ^ab^	0.135 ^b^	0.293 ^a^	0.147 ^b^	0.248 ^b^	0.029	0.099	0.084	0.009
Isobutyrate	0.003 ^c^	0.009 ^ab^	0.003 ^c^	0.011 ^a^	0.003 ^c^	0.009 ^b^	0.003	0.037	0.002	0.024
Butyrate	0.090 ^ab^	0.159 ^ab^	0.086 ^ab^	0.176 ^b^	0.097 ^ab^	0.145 ^a^	0.029	0.338	0.223	0.020
Isovalerate	0.004 ^c^	0.014 ^ab^	0.004 ^c^	0.015 ^a^	0.004 ^c^	0.013 ^b^	0.001	0.031	0.013	0.019
Valerate	0.007 ^c^	0.012 ^ab^	0.007 ^c^	0.013 ^a^	0.007 ^c^	0.011 ^b^	0.001	0.023	0.042	0.008
VFA profile (%)										
Acetate	72.2	66.3	72.4	66.3	71.7	66.5	3.76	0.694	0.392	0.124
Propionate	16.1	19.3	16.2	19.5	16.5	19.6	2.68	0.230	0.487	0.455
Isobutyrate	0.363	0.708	0.367	0.696	0.358	0.696	0.065	0.321	0.065	0.373
Butyrate	10.1	11.8	9.78	11.6	10.2	11.4	2.10	0.512	0.643	0.188
Isovalerate	0.447	1.01	0.447	0.993	0.439	0.991	0.143	0.228	0.112	0.599
Valerate	0.810	0.873	0.815	0.866	0.798	0.845	0.159	0.054	0.834	0.637

^†^ SEM, standard error of the mean (n = 12 for Trt and n = 18 for inoculum sex). ^a–c^ Means within a row with different superscripts differ significantly according to diets and/or sexes at each incubation time (*p* < 0.05).

**Table 5:** Effects of dietary supplementation with *Yucca schidigera* extract (YSE; 300 mg/kg feed), sex and time on faecal dry matter (DM), acid-insoluble ash (AIA), faecal crude protein (CP) concentration and apparent total tract digestibility (ATTD) of CP and organic matter (OM) in fattening pigs during a 42-day in vivo trial (Experiment 2).

	Trt		Sex		SEM ^†^	*p*-Value ^‡^			
	Control	YSE300	Males	Females		Trt	Sex	Time	Trt × Time
DM in faeces (%)									
Day 0	27.1 ^ab^	26.4 ^a^	25.8 ^d^	27.8 ^e^	0.638	0.548	0.006	<0.001	0.916
Day 21	29.8 ^c^	29.5 ^bc^	28.5 ^e^	30.9 ^f^					
Day 42	30.2 ^c^	29.8 ^c^	28.9 ^e^	31.2 ^f^					
AIA in faeces (%)									
Day 0	3.80 ^y^	3.79 ^y^	3.71	3.88	0.092	<0.001	0.099	<0.001	<0.001
Day 21	4.58 ^x^	3.81 ^y^	4.12	4.27 ^x^					
Day 42	4.60 ^x^	3.74 ^y^	4.14	4.20					
CP content in faeces (%)									
Day 0	20.6 ^a^	20.7 ^a^	21.0 ^a^	20.3 ^a^	0.338	0.310	0.627	<0.001	0.229
Day 21	19.1 ^b^	18.2 ^b^	18.7 ^b^	18.6 ^b^					
Day 42	18.2 ^b^	18.0 ^b^	17.9 ^b^	18.3 ^b^					
ATTD of CP (%)									
Day 0	86.2 ^z^	86.1 ^z^	85.6 ^y^	86.7 ^y^	0.350	<0.001	0.109	<0.001	0.023
Day 21	89.4 ^x^	87.8 ^y^	88.4 ^x^	88.8 ^x^					
Day 42	89.9 ^x^	87.7 ^y^	88.9 ^x^	88.7 ^x^					
ATTD of OM (%)									
Day 0	90.6 ^y^	90.6 ^y^	90.4 ^y^	90.8 ^xy^	0.216	<0.001	0.124	0.009	<0.001
Day 21	92.1 ^x^	90.4 ^y^	91.1 ^xy^	91.5 ^x^					
Day 42	92.1 ^x^	90.2 ^y^	91.1 ^xy^	91.2 ^xy^					

^†^ SEM, standard error of the mean (n = 10 for Trt and sex). ^‡^ No interactions were observed between Trt and sex, sex and time, or Trt, sex and time for the variables evaluated (*p* > 0.10).^a,b,c^ Means within a variable differ significantly across sampling time (*p* < 0.05). ^d,e,f^ Means differ significantly between sexes (*p* < 0.05). ^x–z^ Means differ significantly according to the Trt × time interaction (*p* < 0.05).

**Table 6:** Effects of dietary supplementation with *Yucca schidigera* extract (YSE; 300 mg/kg feed) and sex on faecal ammonia concentration, total volatile fatty acids (VFA) and VFA profile measured at day 42 of the in vivo trial in fattening pigs (Experiment 2).

	Trt		Sex		SEM ^‡^	*p*-value ^§^	
	Control	YSE300	Males	Females		Trt	Sex
Ammonia (µmol/g DM)	166	147	146	168	14.2	0.359	0.299
VFA ^†^ content (µmol/g DM)							
Total	308	303	308	304	13.3	0.786	0.824
Acetate	185	183	186	182	8.26	0.855	0.752
Propionate	64.5	63.4	63.5	64.4	3.50	0.828	0.850
Isobutyrate	7.76	7.84	7.70	7.90	0.387	0.884	0.720
Butyrate	32.3	30.5	33.0	29.8	1.90	0.510	0.259
Isovalerate	11.3	11.0	10.7	11.6	0.622	0.769	0.289
Valerate	7.41	7.46	7.17	7.70	0.524	0.946	0.488
VFA profile (%)							
Acetate	60.0	60.4	60.4	60.0	0.737	0.657	0.732
Propionate	20.9	20.9	20.5	21.2	0.577	0.961	0.394
Isobutyrate	2.53	2.60	2.53	2.60	0.104	0.631	0.637
Butyrate	10.49	9.98	10.69	9.78	0.399	0.375	0.128
Isovalerate	3.68	3.65	3.50	3.83	0.192	0.917	0.245
Valerate	2.42	2.46	2.36	2.52	0.146	0.847	0.465

^†^ VFA, volatile fatty acids. ^‡^ SEM, standard error of the mean (n = 10 for Trt and sex). ^§^ No interactions were observed between Trt and sex (*p* > 0.10).

## Data Availability

Data presented in this study are available upon request from the corresponding authors.
